# Dravet Syndrome—The Polish Family’s Perspective Study

**DOI:** 10.3390/jcm10091903

**Published:** 2021-04-28

**Authors:** Justyna Paprocka, Anita Lewandowska, Piotr Zieliński, Bartłomiej Kurczab, Ewa Emich-Widera, Tomasz Mazurczak

**Affiliations:** 1 Department of Pediatric Neurology, Faculty of Medical Sciences in Katowice, Medical University of Silesia, 40-752 Katowice, Poland; marekwidera@wp.pl; 2 Students’ Scientific Society, Department of Pediatric Neurology, Faculty of Medical Sciences in Katowice, Medical University of Silesia, 40-752 Katowice, Poland; anitalewandowska22@gmail.com (A.L.); gchpz@interia.pl (P.Z.); kurczab.bartlomiej@gmail.com (B.K.); 3 Clinic of Paediatric Neurology, National Research Institute of Mother and Child, 01-211 Warsaw, Poland; tomasz.mazurczak@imid.med.pl

**Keywords:** Dravet syndrome, epilepsy, children, quality of life

## Abstract

Aim: The aim of the paper is to study the prevalence of Dravet Syndrome (DS) in the Polish population and indicate different factors other than seizures reducing the quality of life in such patients. Method: A survey was conducted among caregivers of patients with DS by the members of the Polish support group of the Association for People with Severe Refractory Epilepsy DRAVET.PL. It included their experience of the diagnosis, seizures, and treatment-related adverse effects. The caregivers also completed the PedsQL survey, which showed the most important problems. The survey received 55 responses from caregivers of patients with DS (aged 2–25 years). Results: Prior to the diagnosis of DS, 85% of patients presented with status epilepticus lasting more than 30 min, and the frequency of seizures (mostly tonic-clonic or hemiconvulsions) ranged from 2 per week to hundreds per day. After the diagnosis of DS, patients remained on polytherapy (drugs recommended in DS). Before diagnosis, some of them had been on sodium channel blockers. Most patients experienced many adverse effects, including aggression and loss of appetite. The frequency of adverse effects was related to the number of drugs used in this therapy, which had an impact on the results of the PedsQL form, particularly in terms of the physical and social spheres. Intensive care unit stays due to severe status epilepticus also had an influence on the results of the PedsQL form. Conclusions: Families must be counseled on non-pharmacologic strategies to reduce seizure risk, including avoidance of triggers that commonly induce seizures (including hyperthermia, flashing lights and patterns, sleep abnormalities). In addition to addressing seizures, holistic care for a patient with Dravet syndrome must involve a multidisciplinary team that includes specialists in physical, occupational and speech therapy, neuropsychology, social work.

## 1. Introduction

Dravet Syndrome (DS, EIEE6, MIM607208), also known as severe myoclonic epilepsy of infancy (SMEI), was described for the first time by French psychiatrist and epileptologist Charlotte Dravet in 1978. The gene locus for DS is located on chromosome 2q24.3 [1]. Approximately 90% of patients carry a heterozygous de novo mutation. About 75–85% of individuals affected by DS have loss-of-function mutations in the *SCN1A* gene encoding the sodium channel alpha subunit [2]. In *SCN1A* mutation negative patients with clinical similarity to DS, different genetic background is found, particularly the following gene mutations: *PCDH19*, *CHD2*, *STXBP1*, *HCN1*, *GABRG2*, *GABRA1*, and *SCN1B* [3].

The incidence of DS is estimated at between 1:15,700 (in US) and 1:40,900 (in the general population). According to the International League Against Epilepsy (ILAE), DS belongs to the group of developmental and epileptic encephalopathies (DEE) with the prevalence of 1:2000 children and is responsible for 10% of DEE. Decreased function of Nav1.1 sodium channels in GABAergic inhibitory interneurons leads to regression of psychomotor development, refractory epilepsy, and numerous comorbidities (motor, behavioral and cognitive impairment), which may reduce the quality of life of patients and their families [4].

During infancy, the first epileptic seizures (onset between 1 and 18 months) may manifest as febrile or afebrile seizures, hemiclonic or tonic-clonic seizures. With age, polymorphic seizures are observed by the age of 2 years (myoclonic seizures, focal seizures) and atypical absences are seen after the age of 2 years. At onset, brain MRI and psychomotor development are usually normal, and electroencephalography (EEG) does not show abnormalities or results in nonspecific changes. Hyperthermia is the most important triggering factor [5]. Other provoking factors include visual patterns, flashing lights, hot bathing, overexertion, and eating [6]. Intellectual disability is evident by the age of 18–60 months.

There is a natural tendency to decrease epileptic seizures in late childhood and adulthood. Therefore, many researchers divide the clinical course into three stages, i.e., the seizure onset (up to 12 months), the worsening phase (between ages 1 and 5 years), and the stabilization phase (before 10 years of age). During seizures onset period, the first seizures associated with fever, epileptic states appear, while psychomotor development is within normal limits. During the worsening phase, further types of seizures appear, including myoclonic, atypical absence, and focal seizures, around 2nd year of age the psychomotor developmental delay is evident. During the stabilization phase the motor disabilities including crouching, choreoathetosis, pyramidal signs, parkinsonian syndrome are observed [7].

Sudden unexpected death (SUDEP) occurs in approximately 20% of DS patients before the age of 20. The first-line antiepileptic treatment usually consists of valproic acid and clobazam. Stiripentol, cannabidiol, and topiramate are used as second-line treatment [8,9].

Third-line agents include levetiracetam, clonazepam, zonisamide, and ethosuximide. The efficacy of rufinamide, bromides, and acetazolamide remains questionable. Fenfluramine is under study in most European countries [10,11]. Ketogenic diet and vagus nerve stimulation are frequently combined with pharmacotherapy.

In older children and adults, EEG may show background slowing with ictal discharges (generalized spike or polyspike waves, spikes, and waves), and the photoparoxysmal response. Subsequent brain MRI shows hippocampal sclerosis or mild cerebral atrophy [1].

The aim of this study was to present the Polish group of patients with DS, assess their quality of life and evaluate the factors that may affect this quality, such as the time until diagnosis, the treatment schedule before establishing the diagnosis of DS and treatment-related adverse effects.

## 2. Materials and Methods

The survey was emailed to 211 caregivers of patients with DS who were the members of the Polish support group of the Association for People with Severe Refractory Epilepsy DRAVET.PL. 55 (26%) individuals responded and completed the anonymous survey. All participants provided informed consent. The patients ranged from 2 to 26 years of age (mean age 8.891 years, SD ± 5.133 years). All of patients were Polish. Mutation in the *SCN1A* gene was confirmed in all patients fulfilled the clinical criteria of Dravet syndrome. The first part of the study (based on the self-prepared questionnaire) provided the information on the family history, gestation, delivery period, disease course, comorbidities, treatment, and the current condition of patients. In the second part, the respondents were asked to complete the 23-item Pediatric Quality of Life Questionnaire (PedsQL 4.0 Generic Core Scales) related to physical, emotional, social functioning, and school performance within the recent month. Each question was rated on a 5-point scale from 0 to 4 (0—never a problem and 4—almost always a problem). Statistica 13.1 software (TIBCO Software Inc., Palo Alto, CA, USA)was used for statistical analysis. Spearman’s correlation test, Pearson’s Chi-square test and Mann-Whitney U test were performed.

## 3. Results

The study group was comprised of 25 female (45%) and 30 male (55%) patients. The mean maternal age at delivery was 28.78 years (age range 20–39 years).

Abnormal pregnancy was reported in 27.3% of women and included the following conditions: placental abruption, uterine hematoma, arterial hypertension, hemorrhage from the genital tract, premature contractions, serological conflict, umbilical cord wrapping, and gestational diabetes. Delivery at term was reported in 52 patients between 38 and 42 weeks of gestation, 3 patients were premature (born at 31, 34 and 36 weeks of gestation).

In 53 (96.36%) patients, the Apgar score ranged from 8 to 10 (Table 1). The mean birth weight was 3387.9 g. The mean head circumference was 34.64 cm (range 31–38) (Table 1).

The first neurological assessment was performed due to epileptic seizures/status epilepticus (*n* = 14), febrile seizures (*n* = 8), neurodevelopmental disorders (*n* = 3), and abnormal EEG results without the clinical manifestation of epileptic seizures (*n* = 2), prematurity or clubfoot. The first neurological examination showed no abnormalities in most patients (71.1%). Hypotonia or abnormal distribution of muscle tone was found in 7 patients.

### 3.1. The Occurrence of the First Seizure

**Figure 1:**
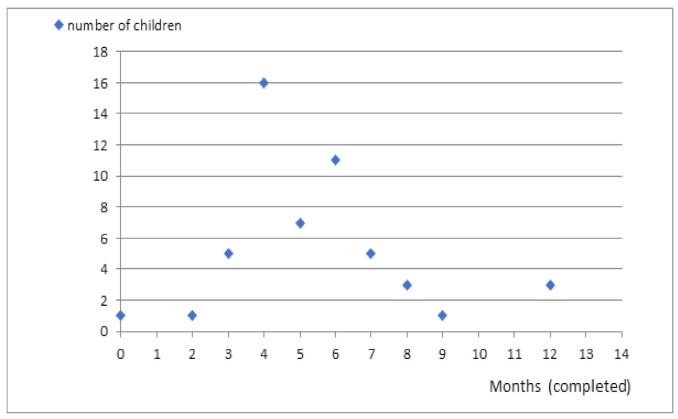
Time of the first seizure.

In our study, 21 (38%) parents indicated that the period after vaccination was the time when the first epileptic seizure occurred. The first attack was reported during fever-associated infection in other 21 (38%) patients. Three (5.5%) caregivers indicated high ambient temperature as a causative factor. The circumstances of the occurrence of the first seizure were not specified in the case of the remaining patients (Figure 1).

### 3.2. Seizure Frequency

Prior to the diagnosis of DS, more than one epileptic seizure per week had been experienced by 56% of the patients (range from 2–3 seizures per week to several hundred seizures per day). Tonic-clonic seizures or hemiconvulsions were found in 52 children, and only 3 patients were seizure-free. In 58% of patients, seizures lasted longer than 30 min. 67% of parents confirmed that drugs such as carbamazepine and lamotrigine exacerbated seizure frequency. Only 5% of patients were seizure-free except for the time of infection.

Prior to the diagnosis of DS, 47/55 (85%) patients had experienced status epilepticus lasting more than 30 min. No response to rectal diazepam or buccal administration of midazolam was observed in 15/55 (27%) patients. In these cases, thiopental and intravenous clonazepam were more effective. 67.3% of patients were hospitalized at the intensive care unit (ICU). The number of hospitalizations ranged from 1 to 12 (29.7%).

### 3.3. Anti-Epileptic Drugs (AEDs) before the Diagnosis of DS

After the second epileptic seizure, treatment was not introduced only in 5 patients. In the remaining group (74%), the first drug was valproic acid initially alone and later in combination with other antiepileptic drugs (AEDs), including levetiracetam (*n* = 7) and phenobarbital (*n* = 3). Other drugs which were introduced after the first seizure included phenobarbital as monotherapy (*n* = 6), carbamazepine as monotherapy (*n* = 3), topiramate (*n* = 3), and nitrazepam (*n* = 1).

Treatment was not effective as reported by 27 respondents. The improvement was “minimal” in 8 patients, and seizures were still present. However, they were either less frequent or occurred later. Five respondents reported that the therapy increased the number of seizures. In 10 children, the antiepileptic treatment was effective (seizure reduction > 75%). Among the medications that parents indicated as working minimally or effectively were valproic acid and levetiracetam.

### 3.4. Time to Diagnosis

**Figure 2:**
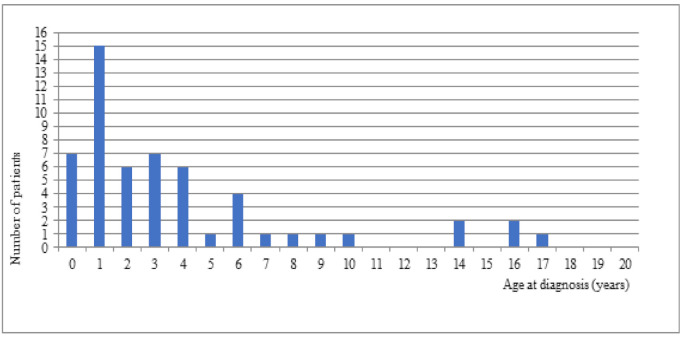
Age at diagnosis.

In our study, 46 patients (83.63%) were diagnosed in the first 6 years of life (Figure 2). The diagnosis was established within first 12 months of age in 7 patients (18.9%) (Figure 2). The time to diagnosis did not have a statistically significant effect on the PedsQL scores. No significant correlation was found between the specific spheres of patients’ lives and the increased PedsQL scores (*p* = 0.94). There have been changes in the acceleration of the time of DS diagnosis in the latest years with more advanced genetic tools.

### 3.5. Antiepileptic Drugs (AEDs) after the Diagnosis of DS

After establishing the diagnosis, 53 patients remained on polytherapy (drugs recommended in DS). About 85% of patients were given valproic acid or sodium valproate, 67% stiripentol, 35% clobazam, 33% levetiracetam, 31% topiramate, and 16% potassium bromide. Cannabidiol was used in 12% of patients, while clonazepam and phenobarbital in 3% of subjects. Other AEDs included ethosuximide, zonisamide, nitrazepam, carbamazepine, and pregabalin. Valproic acid alone was used only in 2 patients. Stiripentol resulted in termination of status epilepticus in 38 patients.

The group of patients with a history of ICU stay chronically used a statistically significantly higher number of drugs compared to the group of patients without a history of hospitalization at the ICU.

### 3.6. Non-Pharmacological Treatment

Non-pharmacological treatment methods for epileptic seizures were reported by 18 respondents. Among non-invasive methods ketogenic diet (*n* = 10), cannabidiol oil (*n* = 2), non-specified elimination diet, carnosine treatment, and homeopathy were reported. Other therapeutic methods, invasive, were vagus nerve stimulation (*n* = 5) or callosotomy (*n* = 1).

### 3.7. Adverse Effects of AEDs

Combination therapy and permanent change in the treatment regimen were associated with the possible occurrence of adverse effects. The caregivers were often unable to specify which medication was responsible for the occurrence of adverse effects. Table 2 includes the most common symptoms as reported by the caregivers.

**Figure 3:**
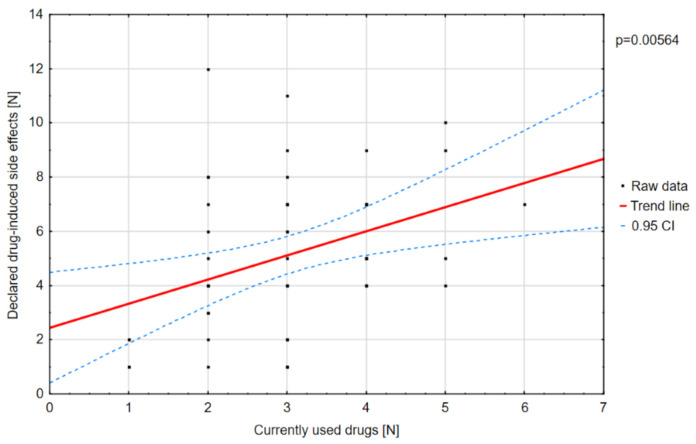
Reported drug-induced adverse effects depending on the number of drugs used.

**Figure 4:**
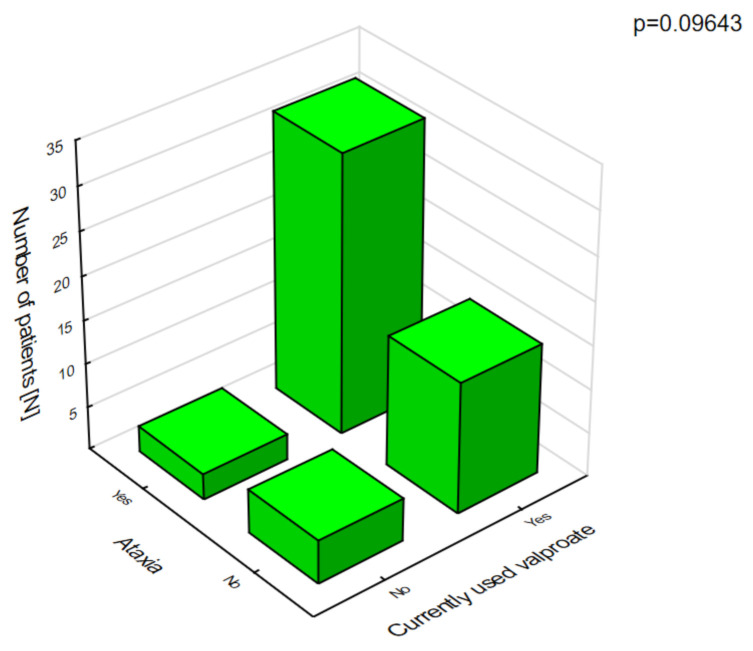
Valproic acid and ataxia.

**Figure 5:**
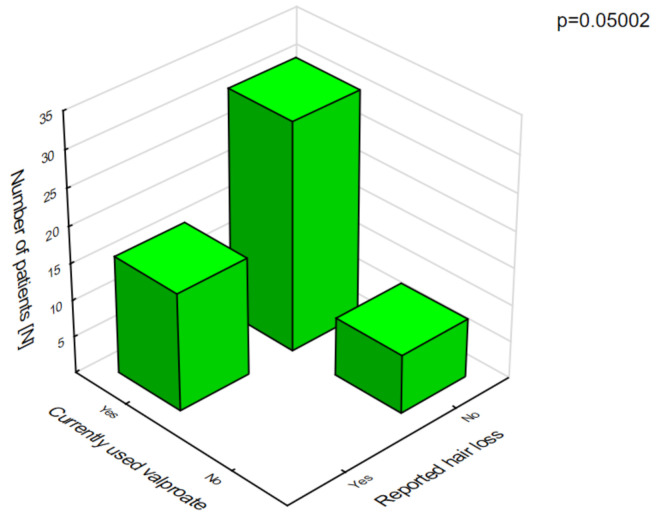
Valproic acid and hair loss.

Polytherapy is a significant factor for the incidence of adverse effects and lowering the quality of life (Figure 3). The analysis of the adverse effects reported by the caregivers showed that different agents often had the same adverse effects, which may be related to their combination in therapy. Valproic acid was the most common drug in our patients with DS [8]. The most frequent adverse effects of this drug were associated with a statistically significant increase in ataxia (Figure 4) and hair loss (Figure 5).

**Figure 6:**
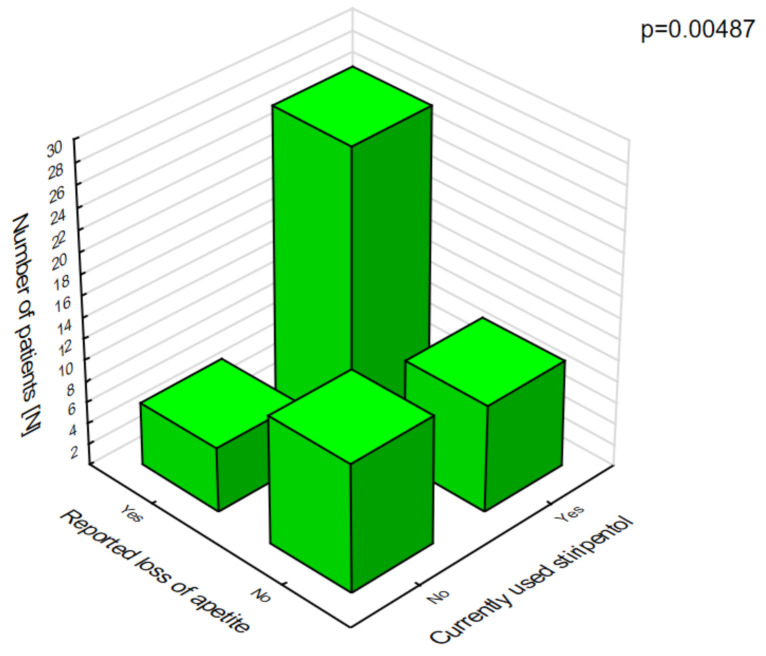
Stiripentol and loss of appetite.

**Figure 7:**
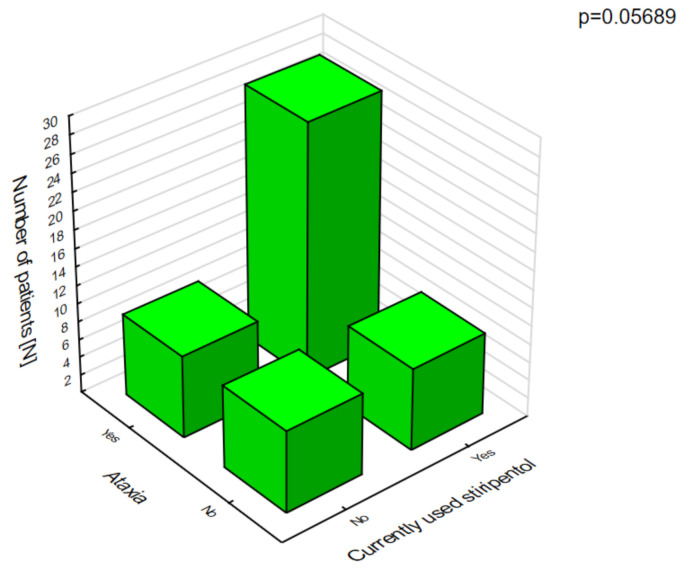
Stiripentol and ataxia.

Stiripentol was statistically significantly more likely to cause anorexia nervosa (Figure 6), which was associated with weight loss and ataxia (Figure 7).

**Figure 8:**
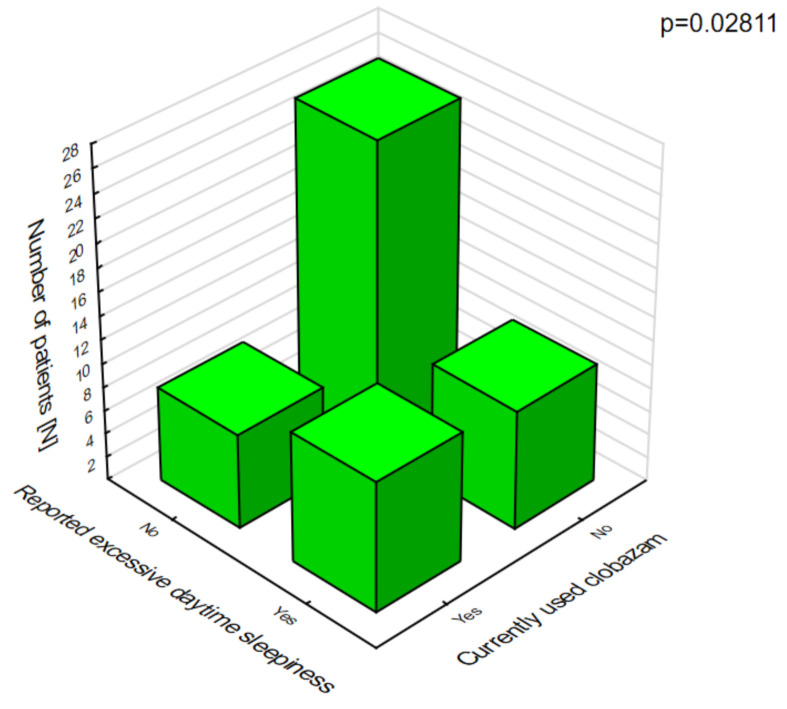
Clobazam and daytime sleepiness.

**Figure 9:**
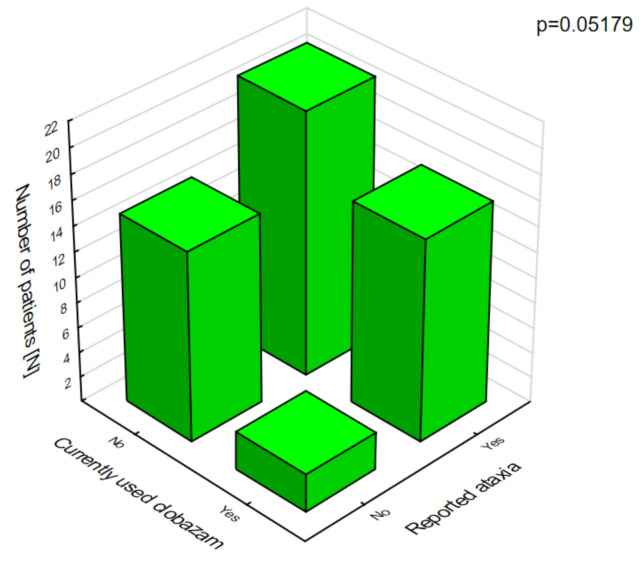
Clobazam and ataxia.

In patients on clobazam, excessive daytime sleepiness (Figure 8) and ataxia (Figure 9) occurred significantly more often compared with patients who were not given this drug.

### 3.8. Comorbidities

The most common comorbidities in DS are given in Table 3.

### 3.9. The Degree of Disability

According to the psychological assessment, patients presented with moderate (31%), severe (11%), mild (10%), or profound (2%) disability. Other patients did not present disability (Table 4).

**Figure 10:**
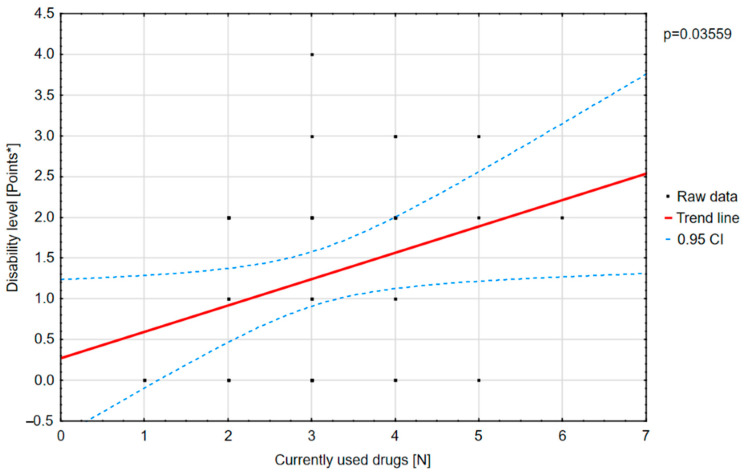
Correlation between the drugs used and the degree of disability.

A positive correlation was found between the number of drugs and the degree of disability (Figure 10).

### 3.10. Analysis of the PedsQL Generic Score (4.0)

The answers were related to the most recent month of children’s lives. The results were collected according to the forms assigned to specific age groups so that various aspects of life were adequate for their age. The scale reflecting the occurrence of the problem was as follows:

0: never

1: almost never

2: sometimes

3: often

4: almost always

#### 3.10.1. Physical Functioning

**Figure 11:**
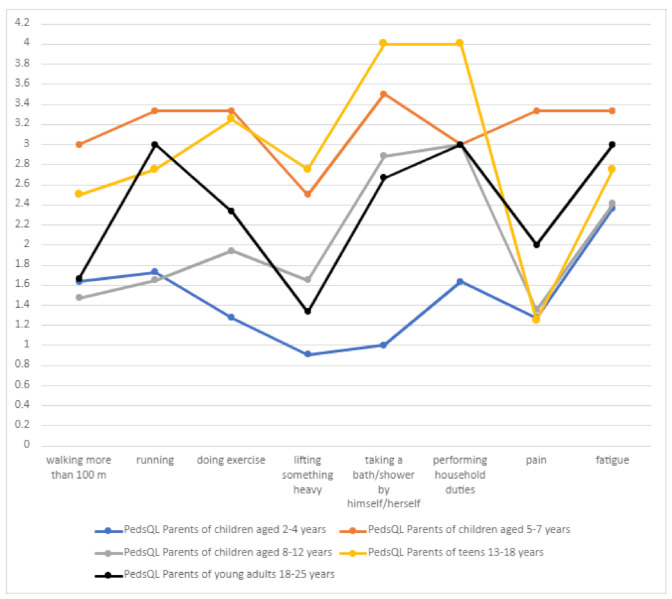
Physical functioning in children with Dravet syndrome.

In terms of the physical sphere, the independence of DS patients was a particularly important problem (often or almost always related to taking a bath/shower or performing household chores) (Table 5, Figure 11). In the age group of 5–7 years, the problem related to walking more than 100 m and running significantly increased, which may be connected with seizure intensity in this age group. In the group of 13–18 years, the problem associated with walking and running could worsen ataxia.

There was a statistically significant positive correlation between the number of drugs used and the scores on the “physical functioning” component of the PedsQL scale.

#### 3.10.2. Emotional Functioning

**Figure 12:**
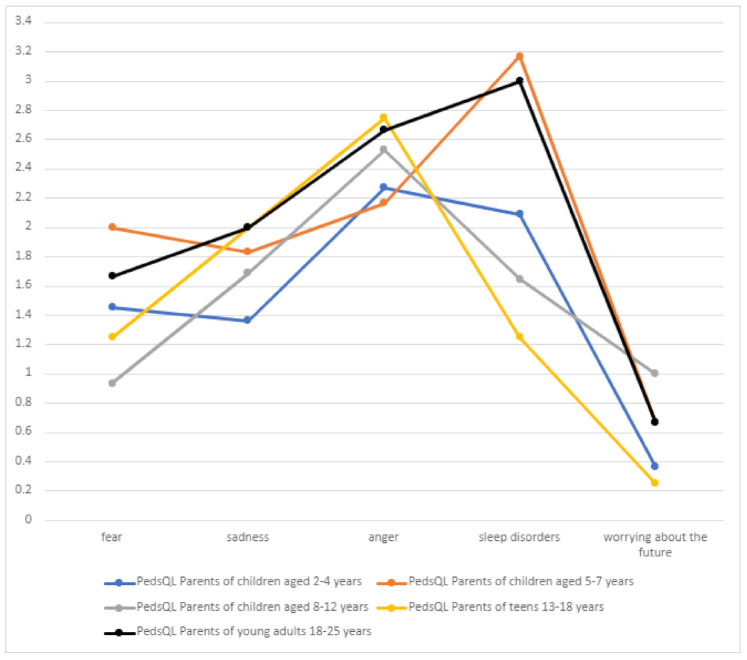
Emotional functioning of children with Dravet syndrome.

Significant problems related to the emotional sphere included the feeling of anger and sleeping problems, which are inextricably linked to DS and should be of interest to attending physicians (Table 6, Figure 12). Worrying about the future was the least frequent problem (mean score 0.589) in all patient groups, which may be due to the child’s age or intellectual disability.

#### 3.10.3. Social Functioning

**Figure 13:**
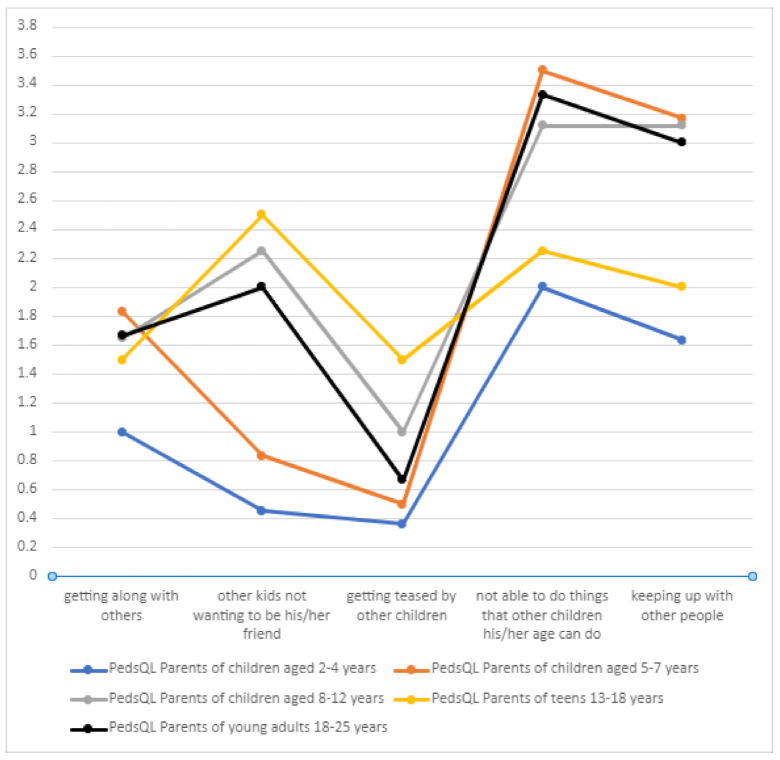
Social functioning of DS patients.

In terms of the social sphere, DS patients very often could not perform the activities their peers did in certain age groups (Table 7, Figure 13). Getting teased by other children was not a significant problem in the DS group, which may be due to the care provided by families and special facilities that the children attended. As in the case of the physical domain, a positive correlation was found between the number of treatment-related adverse effects and the score on the “social functioning” component of the PedsQL scale (*p* = 0.00536).

#### 3.10.4. School Functioning

**Figure 14:**
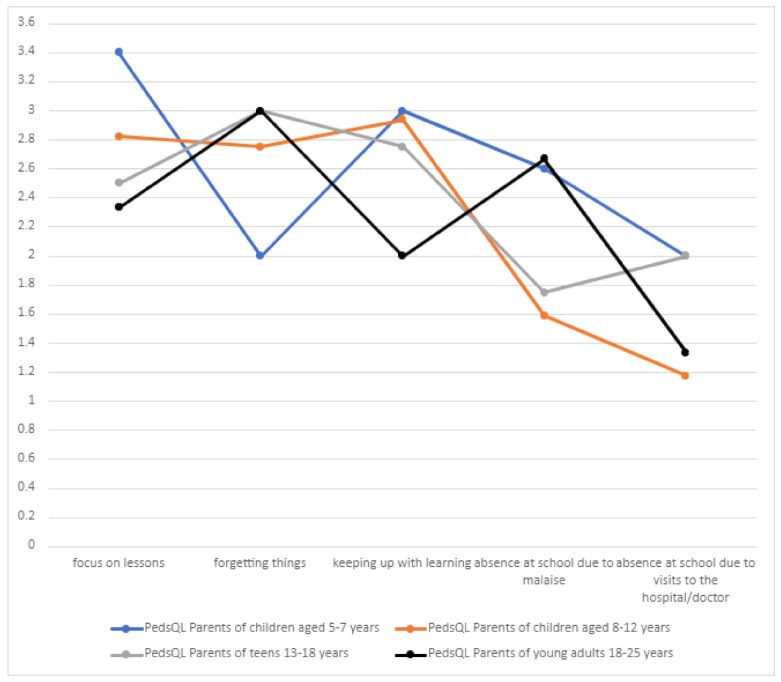
School functioning in children with DS.

School functioning among patients was related to their present condition and individual skills. The main problems related to school functioning included the intellectual sphere (focusing on lessons, forgetting things, and keeping up with learning) (Table 8, Figure 14).

## 4. Discussion

The clinical diagnosis of Dravet syndrome is supported by the presence of abnormalities in the sodium channel gene *SCN1A* (found in 75% of cases). Over 150 mutations in the *SCN1A* gene have been described. Another form of epilepsy connected with *SCN1A* mutations, apart from DS, is epilepsy with febrile seizures plus and epilepsy of infancy with migrating focal seizures [1,2,3,4]. Familial hemiplegic migraine also may be caused by abnormalities within *SCN1A* gene [1,2,3,4].

In our study, 38% of parents were aware about the connection between the first seizure and the preceding vaccination (diphtheria-tetanus-pertussis). Tro-Baumann et al., Wong et al., and Desnous et al. [12] reported that vaccination could be a triggering factor for epileptic seizures in 50% of DS patients [13]. According to the expert panel, prophylactic administration of ibuprofen before and several days after immunization may minimalize the risk of seizures. Based on retrospective data, immunization did not affect the occurrence of seizures, the degree of seizure control, or the evolution of comorbidities. Auvin et al. [14] found that the levels of interleukin (IL)-6, IL-1, and tumor necrosis factor (TNF) alpha were increased in DS, whereas the level of IL-10 was decreased. Interestingly, based on 12 DS patients, a shift toward an M1 pro-inflammatory phenotype of monocytes in DS was also reported. The same pro-inflammatory phenotype of monocytes is predominately induced by acute pathological conditions, including stroke, traumatic brain injury, or systemic inflammation.

Warner et al. [15] demonstrated the occurrence of epileptic seizures induced by temperature elevation in mouse models with the *SCN1A* mutation. It was shown that an increase in temperature resulted in myoclonic seizures, generalized tonic-clonic seizures, and increased anxiety symptoms. In addition, myoclonic seizures were more severe in younger mice than in older mice, which was related to hypersensitivity to temperature elevation in younger DS patients [16] and resolution of myoclonic seizures in adults.

A very interesting conclusion was found in a retrospective study of patients with epileptic seizures and the *SCN1A* gene mutation. According to Nabbout et al. [17], in the group of 20 patients (12 girls, 8 boys) the mean age at diagnosis was 2 years and 10 months. The results varied from country to country (Italy—1 year 1 month, Australia—2 years, Great Britain—4 years 2 months, and the USA—5 years). Cetica et al. [18] showed that the age of onset of seizures could lead to the diagnosis of DS. The risk of DS was 85% in patients with first seizures (generalized tonic-clonic—71%, partial seizures—19%, myoclonic seizures—4%) before 6 months of age, 51% at 6–12 months, and 0% after 12 months. In their study, Gataullina et al. [16] also indicated that the first seizures (i.e., tonic-clonic, or partial) occurred in the first year of life, usually between 4 and 8 months of age. The diagnosis of DS can be made according to the criteria [19] proposed by epileptologists who together with parents (members of the American Dravet Syndrome Foundation) developed a consensus to facilitate the diagnosis of DS based on 9 clinical features, including the characteristic onset of tonic-clonic or partial seizures during the first year of life. In our study of 55 DS patients, the maximum age at which the first seizure occurred was 12 months. In our study, 46 (83.63%) patients were diagnosed within the first 6 years of life, and 7 (18.9%) patients were diagnosed before 12 months of age. The mean age at diagnosis was 3.85 years. It is possible to compare the situation in Poland to other countries.

Lagae et al. [20] assessed the quality of life in a multinational cohort based on a survey of DS caregivers from different European countries (85% children and 17% adults). Their survey showed that the diagnosis had been made in 88% of children within the same year of initial presentation (until 12 months of age). In older children and adults, the diagnosis of DS was made within 4 years after the first seizure in 20% and 83% of patients, respectively.

The early diagnosis is crucial to the management of DS because it allows for avoiding unnecessary therapies based on sodium channel, exacerbating seizures, or prolonged status epilepticus. It also allows to decrease the impact of factors, which may induce sudden unexpected death in epilepsy (SUDEP), such as lack of treatment with antiepileptic drugs, frequent medication changes, increased seizure frequency. It is believed that SUDEP may be caused by various factors and its etiology is not uniform. Among others, a subsequent bradycardia after a generalized epileptic seizure has been implicated as a cause [21]. Early diagnosis, and thus appropriate treatment, allows seizures to be controlled and thus indirectly reduces the risk of SUDEP.

From the neurological perspective, the better antiepileptic control, the better the neuropsychological outcome. Optimal management of epilepsy in DS is still limited. Febrile convulsions before the onset of epileptic seizures occurred in less than 50% of DS patients. Damiano et al. [13] concluded that sequencing the *SCN1A* gene in infants with prolonged febrile seizures could facilitate faster diagnosis of DS. Researchers agreed that the early diagnosis and treatment could improve the prognosis of long-term outcomes. Therefore, the recommendations from a North American Consensus Panel give a new insight into these problems.

An early diagnosis of DS is crucial for adequate treatment. Antiepileptic drugs which worsen seizures (including the increase in frequency and duration of seizures) include carbamazepine and Phenobarbital [10]. These drugs had been commonly used in antiepileptic therapy in our patients before the diagnosis of DS was confirmed. Six patients were treated with phenobarbital and 3 subjects with carbamazepine alone.

Branklaus et al. [22] demonstrated the usefulness of genetic diagnosis in patients with early childhood epilepsy and confirmed the positive effect of early confirmation of DS on the change in appropriate antiepileptic treatment in these patients. 87% of the respondents appreciated the diagnosis of the *SCN1A* gene mutation, which allowed 74% of patients (91/123) to receive appropriate treatment and avoid treatment that is not recommended in DS (sodium channel blockers such as carbamazepine).

Prior to the diagnosis of DS, in the Norwegian population, up to 38% of patients used sodium channel blockers. After the diagnosis, only 6 of the 55 patients were on sodium channel blockers. A study including 274 patients with DS from several European countries [23] showed that 33% of patients were on sodium channel blockers before the diagnosis of DS.

Also this study [23] showed that only 5% of patients were on monotherapy. The regimen of AEDs in DS in Poland and other European countries was similar. Stiripentol was more common among Polish patients compared to other European patients (67% vs. 42%). Across the age groups, the use of valproic acid and clobazam was the most common [24]. For DS, three trials with cannabidiol (Epidiolex) were completed (Devisky et al. 2017, Devinsky et al. 2019, Miller et al. 2019). It was approved by the Food and Drug Administration (FDA) in 2018 and the European Medicines Agency (EMA) in 2019 as add-on antiepileptic therapy for DS patients over two years of age. Epidiolex may be imported to Poland with the consent of the Ministry of Health. Respondents reported that potassium bromide [25] was also used in polytherapy in several patients. The treatment scheme without benzodiazepines and with the use of potassium bromide at later stages was proposed by Gutaullina and Dulac in 2016 [16].

To conclude our study, we asked respondents to list all AEDs that patients had ever used and to select those that were currently used. A total of 16 AEDs had been used by more than 5% of patients in addition to the ketogenic diet. The most common drug was levetiracetam, which was used by more than 50% of patients at some point of treatment.

Heger et al. [26] in their study on the Norwegian population of 55 patients with DS also noted the problem of polytherapy and the resulting drug–drug interactions and indicated the three most commonly used drugs (i.e., valproic acid, clobazam and stiripentol), which could influence each other’s pharmacokinetics. Stiripentol is a potent inhibitor of hepatic enzymes and can displace valproic acid and clobazam from their protein binding sites, which results in increased drug concentrations in the blood and adverse effects such as ataxia or hair loss. Myers et al. identified the most commonly reported adverse effects in a 12-year follow-up study of 41 patients that evaluated the efficacy of stiripentol in reducing seizures and its combination in therapy with other drugs. [27] Stiripentol was used in combination with valproic acid (71%) and clobazam (80%). The researchers reported anorexia nervosa and weight loss in 49% of patients compared to 60% of our patients affected with anorexia nervosa. Inoue et al. [28] found that the most common adverse effects of stiripentol included drowsiness (39%) and loss of appetite (25%), which were significantly more common in our patients (40% and 60%, respectively). Chiron et al. [29] in their study on the efficacy of stiripentol in 41 patients also reported similar adverse effects (loss of appetite, drowsiness) in 51% of patients on stiripentol, valproic acid, and clobazam. Additionally, these adverse effects resolved in 57% of patients after the dose of stiripentol was reduced. While dealing with the side effects of stiripentol, many of them are also due to valproic acid, because frequently these two drugs are used together (as in our study).

In our study, we showed a weak positive correlation between the age at diagnosis and the degree of disability. The r-Pearson correlation was 0.401 with a significance level of 0.003. Lagae et al. [20] found no correlation between the late diagnosis and the occurrence of comorbidities. Their study was comprised of 584 patients, which allowed to achieve more reliable results. Additionally, Conolly et al. [30] showed no significant correlation between the Intelligence Quotient (IQ) and the time of the first seizure and the number and morphology of epileptic seizures. None of the 21 children reached normal values at the age of 6 in the Wechsler Intelligence Scale for Children (WISC) or the Vineland Adaptive Behavior Scale and only 5 children had an IQ of over 60. The IQ was normal before the age of 2. However, it decreased after 3 years of age. The IQ was lower in patients with a mutation in the *SCN1A* gene, which allowed us to conclude that the degree of intellectual disability and the outcome of the study were influenced by other factors, such as the age of patients below 3 years (the age limit for regression), individual variability and the mutation type. A positive correlation between the number of drugs and the degree of intellectual disability may be due to a greater degree of intellectual disability resulting from a greater number of seizures and epileptic conditions [31,32]. Polytherapy, which was necessary in some patients, resulted in treatment-related adverse effects, which affected cognitive abilities [33,34].

As many as 71.1% of patients experienced movement problems. In our study, parents also indicated in the PedsQL form that there were frequent or very frequent problems with performing more complicated movement activities. Based on the PedsQL form, the problem with walking and running increased with age. Conolly et al. [30] indicated the occurrence of problems in the motor sphere, which exacerbated with age. Crouch gait is a significant problem, as indicated by many studies. Up to 50% of patients between 6 and 12 years of age are affected, whereas older patients are additionally affected by the symptoms of Parkinson disease, which significantly reduces the walking ability of DS patients. High prevalence of ataxia is an important issue that influence movement ability of DS patients. Our questionnaire showed that 65% of patients were affected with ataxia. Villas et al. [35] showed that among 225 patients from various countries, 65% presented with ataxia, which was particularly severe in the younger children. Our findings are in line with these observations. Ataxia poses a risk of fall, hinders walking, and requires constant supervision by caregivers.

38% of our patients presented with aggressive behaviors. Using the PedsQL form, parents assessed them as frequent or very frequent without a significant increase in aggressive behaviors in a particular age group. Additionally, Lagae et al. [20] showed that behavioral disorders unrelated to autism or ADHD were found in 51% of patients. Patients may require additional pharmacotherapy or even psychiatric treatment, which may lead to significant drug interactions. In addition, aggressive behavior, which can be an adverse effect of some AEDs, may result in drug discontinuation. It is related to poorer control of epileptic seizures. Aggressive behavior is considered a factor reducing the quality of life.

Sinoo et al. [36] found that 55.6% of DS patients were affected by behavioral disorders. In a group of 116 patients, the most frequent problem was related to concentration disturbances. Among our patients, the parents indicated frequent or very frequent problems with remembering new things, learning, and attention during classes.

Sleep disorders, which significantly reduce the quality of life, are inextricably linked with DS. They occurred in our patients mainly in the group of young children below the age of 7 years and in young adults. Licheni et al. [37] showed that 75% of patients with DS reported sleep disorders and 53% of them had night seizures. As many as 39% of patients required hypnotic drugs. Melatonin [38] (administered to our patients), medical cannabis, or psychiatric drugs such as clonidine or fluoxetine were used. In comparison, a study of patients with juvenile myoclonic epilepsy treated with levetiracetam observed that reduced sleep quality and daytime sleepiness increased the risk of seizures despite adequate antiepileptic treatment [39]. Adequate sleep hygiene is therefore one of the factors that play a major role in the success and efficacy of treatment [40].

Brunklaus et al. [41] evaluated health-related quality of life (HRQOL) in 125 patients with the phenotype of DS with the *SCN1A* mutation. The assessment was based on the Impact of Pediatric Epilepsy Scale (IPES), the Epilepsy & Learning Disabilities Quality of Life Questionnaire (ELDQOL), the Strengths and Difficulties Question (SDQ), and PedsQL. Hyperactivity and short attention span were strong predictors of poor HRQOL. Other factors connected with worse HRQOL included early onset of epilepsy, epilepsy severity and myoclonic seizures. In our study, parents did not indicate frequent problems with children’s peers in the sphere of social functioning, which could be due to the specificity of the facilities attended by patients. SDQ showed the highest scores related to behavioral problems, hyperactivity/inattention, and peer relationships. However, Brunklaus et al. did not find any correlation between the incidence of behavioral disorders and a specific age group. In turn, Lagae et al. [20] reported an increase in behavioral problems with age in DS children and a slight decrease or stable prevalence of behavioral disorders in adult patients with DS.

In a survey paper by Villas et al. [35], 256 responses were obtained, where common problems in DS other than seizures were listed. In the questionnaire, caregivers frequently reported behavioral and psychiatric problems. It was difficult to measure other symptoms such as anxiety, self-esteem, and depression. Diagnosis of ADHD was the most common in the 7–10 year age group (38% and 39%, respectively), with the mean prevalence of 29% in patients older than 3 years of age. Other psychiatric symptoms occurred mainly in patients older than 3 years, although they were not so common. In our population, parents reported a frequent problem with aggression in children of all age ranges (38% of patients).

Dorris et al. [31] in their study involving 22 patients with electrical status epilepticus in sleep (ESES) found that it was associated with progressive regression of intellectual abilities. In addition, Meldrum and Brierley [32] reported that seizures in baboons induced with bicuculline (GABA receptor antagonist) lasted from 82 to 299 min.

Eighty-five percent of the subjects in our study had an epileptic episode which lasted longer than 30 min and 67.3% were admitted to the ICU due to this condition. A higher incidence of admission to the ICU indirectly indicated a higher incidence of severe epilepsy and difficulty to control it, which, considering the above studies, may be reflected in lower IQ scores and thus poorer school performance.

The limitation of our study is a small sample of DS patients that is not representative for the Polish population of patients with DS. The caregivers of DS patients live under enormous stress, which may have influenced the questionnaire results. Moreover, the PedsQL questionnaire is a subjective assessment of parents. Of note, it is difficult to draw clear conclusions about the assessment of individual variables included in the questionnaire due to the heterogeneous and often progressive course of the disease in the first years of life as well as individual variability and psychological changes during adolescence.

## 5. Conclusions

Families must be counseled on non-pharmacologic strategies to reduce seizure risk, including avoidance of triggers that commonly induce seizures (including hyperthermia, flashing lights and patterns, sleep abnormalities). In addition to addressing seizures, holistic care for a patient with Dravet syndrome must involve a multidisciplinary team that includes specialists in physical, occupational and speech therapy, neuropsychology, social work.

## Figures and Tables

**Table 1:** Median and IQR of the parameters.

Parameter	Males *N* = 30 (55%)	Females *N* = 25 (45%)
Age of patients [years]	9 ± 5	11 ± 9
Apgar score [points]	10 ± 1	9 ± 1
Birth weight [g]	3527.5 ± 700	3380 ± 690
Birth head circumference [cm]	35 ± 3	33.5 ± 1.5
Mother’s age at birth [years]	35 ± 3	33.5 ± 1.5
Total score in PedsQL [points]	46.5 ± 22	52 ± 16

**Table 2:** Adverse effects during the antiepileptic therapy.

Adverse Effects	Percentage of Patients (*n* = 55)
Ataxia	65
Loss of appetite	60
Hypotonia	60
Psychomotor agitation	49
Drooling	47
Aggressive behavior	38
Excessive daytime sleepiness	40
Insomnia	38
Hair loss	31
Constipation	25
Increased appetite	13

**Table 3:** Comorbidities in DS patients.

Comorbidities	Percentage of Patients (%)
Intellectual disability	54
Hypothyroidism	5
Polycystic ovary syndrome	2
Immune thrombocytopenia	4
Atopic dermatitis	2
Asthma	4
Food allergy	4
Thrombophilia	2
Vasculitis	2

**Table 4:** Intellectual functioning of patients with Dravet syndrome.

Intellectual Disability	Percentage of Patients
Absence of intellectual disability	46
Mild	10
Moderate	31
Severe	11
Profound	2

**Table 5:** Physical functioning in children with Dravet syndrome.

	PedsQL Parents of Children Aged 2–4 Years	PedsQL Parents of Children Aged 5–7 Years	PedsQL Parents of Children Aged 8–12 Years	PedsQL Parents of Teenagers Aged 13–18 Years	PedsQL Parents of Young Adults Aged 18–25 Years
Walking more than 100 m	1.6363	3	1.4705	2.5	1.666
Running	1.72727	3.333	1.647	2.75	3
Doing exercise	1.2727	3.333	1.9411	3.25	2.333
Lifting something heavy	0.909	2.5	1.647	2.75	1.333
Taking a bath/shower by himself/herself	1	3.5	2.8823	4	2.666
Performing household duties	1.6363	3	3	4	3
Pain	1.2727	3.333	1.3529	1.25	2
Fatigue	2.3636	3.333	2.41176	2.75	3

**Table 6:** Emotional functioning in children with Dravet syndrome.

	PedsQL Parents of Children Aged 2–4 Years	PedsQL Parents of Children Aged 5–7 Years	PedsQL Parents of Children Aged 8–12 Years	PedsQL Parents of Teenagers Aged 13–18 Years	PedsQL Parents of Young Adults Aged 18–25 Years
Fear	1.454545	2	0.9375	1.25	1.666
Sadness	1.363636	1.8333	1.6875	2	2
Anger	2.272727	2.16666	2.5294	2.75	2.666
Sleep disorders	2.09090	3.16666	1.647	1.25	3
Worrying about the future	0.363636	0.66666	1	0.25	0.666

**Table 7:** Social functioning of DS patients.

	PedsQL Parents of Children Aged 2–4 Years	PedsQL Parents of Children Aged 5–7 Years	PedsQL Parents of Children Aged 8–12 Years	PedsQL Parents of Teenagers Aged 13–18 Years	PedsQL Parents of Young Adults Aged 18–25 Years
Getting along with others	1	1.8333	1.647	1.5	1.6666
Other kids not wanting to be his/her friend	0.454545	0.83333	2.25	2.5	2
Getting teased by other children	0.363636	0.5	1	1.5	0.6666
Not able to do things that other children his/her age can do	2	3.5	3.11764	2.25	3.333
Keeping up with other people	1.6363	3.16666	3.11764	2	3

**Table 8:** School functioning of patients with DS.

	PedsQL Parents of Children Aged 5–7 Years	PedsQL Parents of Children Aged 8–12 Years	PedsQL Parents of Teenagers Aged 13–18 Years	PedsQL Parents of Young Adults Aged 18–25 Years
Focus on lessons	3.4	2.8235	2.5	2.3333
Forgetting things	2	2.75	3	3
Keeping up with learning	3	2.9375	2.75	2
Absence at school due to malaise	2.6	1.58823	1.75	2.666
Absence at school due to medical visits to the doctor/hospital	2	1.1764	2	1.333

## Data Availability

The datasets generated during and/or analyzed during the current study are available from the corresponding author on reasonable request.
